# Supplementary material for: Development Features of Ixodes ricinus × I. persulcatus Hybrids under Laboratory Conditions
Source: Microorganisms. 2023 Sep 7;11(9):2252. doi: 10.3390/microorganisms11092252 (PMC10536943; doi:10.3390/microorganisms11092252)
Supplement: Supplementary file 1 [file microorganisms-11-02252-s001.zip › Supplementary material.pdf]

Table S1. Duration of feeding, oviposition and hatching of the larvae of *Ixodes ricinus, Ixodes persulcatus,* and hybrid females after mating with the males of various species

Species of female	Species of male	Feeding in March, April				Feeding in June, July				Feeding in September, October			
		Weight of female, mg	Duration of feeding, days	Days from engorgement till Ov	Days from Ov till the start of LL	Weight of female, mg	Duration of feeding, days	Days from engorgement till Ov	Days from Ov till the start of LL	Weight of female, mg	Duration of feeding, days	Days from engorgement till Ov	Days from Ov till the start of LL
I.pers	*I.pers*	348.2±82.0<br>(n=10)	7.9±0.7<br>(n=17)	7.8±1.6 [b]<br>(n=12)	34.8±2.2<br>(n=11)	293.7±59.2 [a]<br>(n=7)	8.5±1.2<br>(n=15)	5.3±1.3 [b]<br>(n=13)	35.4±8.1<br>(n=10)	361.4±61.9 [a]<br>(n=11)	8.4±1.4<br>(n=11)	6.9±1.1<br>(n=11)	37.7±5.9<br>(n=11)
I.pers	*I.ric*	312.2±60.3<br>(n=9)	8.0±0.9 [i]<br>(n=13)	9.2±2.3 [g]<br>(n=11)	41.5±5.0 [k]<br>(n=11)	346.4±45.4<br>(n=9)	8.9±0.3 [i]<br>(n=10)	5.4±1.2 [g]<br>(n=10)	40.9±5.5 [l]<br>(n=9)	380.5±61.5<br>(n=4)	9.0±1.7<br>(n=4)	7<br>(n=4)	57.0±2.8 [k,l]<br>(n=2)
I.pers	Hybrid (♀*I.pers* ×♂*I.ric*)	-	-	-	-	-	-	-	-	267.0±236.2<br>(n=2)	11.5±2.1<br>(n=2)	6<br>(n=1)	47<br>(n=1)
I.ric	*I.ric*	284.3±46.8<br>(n=6)	7.4±0.7 [d]<br>(n=16)	9.7±2.2 [e]<br>(n=15)	36.8±2.4 [j]<br>(n=15)	219.1±74.5 [c]<br>(n=13)	9.4±1.8 [d]<br>(n=19)	7.2±2.0 [e,f]<br>(n=17)	34.9±6.7 [h]<br>(n=17)	355.8±93.9 [c]<br>(n=12)	9.0±2.5<br>(n=12)	10.3±2.5 [f]<br>(n=11)	45.9±2.4 [j,h]<br>(n=11)
I.ric	*I.pers*	287.8±54.7<br>(n=11)	7.7±1.6 [m]<br>(n=20)	12.2±2.5<br>(n=20)	38.4±36<br>(n=19)	258.5±63.5<br>(n=13)	9.5±1.2 [m]<br>(n=13)	10.6±2.8<br>(n=8)	38.6±4.9<br>(n=5)	-	-	-	-
I.ric	Hybrid (♀*I.pers* ×♂*I.ric*)	-	-	-	-	-	-	-	-	217.0±74.6<br>(n=4)	11.3±2.4<br>(n=4)	8.0±0.8<br>(n=4)	56.0±1.4<br>(n=2)
Hybrid (♀*I.pers* ×♂*I.ric*)	*I.ric*	340±17.4<br>(n=3)	7.7±0.6<br>(n=3)	12.5±9.2<br>(n=2)	-	-	-	-	-	-	-	-	-
Hybrid (♀*I.pers* ×♂*I.ric*)	Hybrid (♀*I.pers* ×♂*I.ric*)	153.3±110.8<br>(n=6)	14.3±3.1<br>(n=6)	10.0±1.4<br>(n=2)	-	-	-	-	-	172.3±75.8<br>(n=7)	12.9±3.9<br>(n=7)	9.4±4.1<br>(n=7)	55.0±7.1<br>(n=2)
Hybrid (♀*I.ric* × ♂*I.pers*)	*I.pers*	-	-	-	-	-	-	-	-	76.5±68.6<br>(n=2)	12<br>(n=2)	-	-
Hybrid (♀*I.ric* × ♂*I.pers*)	Hybrid (♀*I.ric* × ♂*I.pers*)	-	-	-	-	-	-	-	-	35<br>(n=1)	12<br>(n=1)	-	-

"-" no data; mean ± SD is presented where applicable; *I.ric – Ixodes ricinus*; *I.pers – Ixodes persulcatus;* Ov – start of the oviposition; LL – start of larvae hatching; superscript letters indicate statistically significant differences between groups according to Mann-Whitney test ($p<0.05$)

Table S2. The duration of feeding and molting of larvae and nymphs of *Ixodes ricinus*, *Ixodes persulcatus*, and their hybrids under laboratory conditions

Species	Phase	Feeding in March, April, May		Feeding in June, July		Feeding in September		Feeding in October		Feeding in November		Feeding in December	
		Feeding duration, days	Days from engorgement till the start of molting[#]	Feeding duration, days	Days from engorgement till the start of molting	Feeding duration, days	Days from engorgement till the start of molting	Feeding duration, days	Days from engorgement till the start of molting	Feeding duration, days	Days from engorgement till the start of molting	Feeding duration, days	Days from engorgement till the start of molting
I. pers	LL	3	28.5±7.8 [a] (n=5)	3	42.3±8.5 [a] (n=6)	3	105.3±11.6 [a,j,h] (n=7)	3	96.0±10.0 [a] (n=3)	-	-	-	-
	NN	3	43.3±0.5 [b] (n=8)	3	32.8±1.5 [b] (n=7)	3	42.8±9.9 [b] (n=5)	3	83.7±5.8 [b,l] (n=4)	3	94.5±0.7 [b] (n=2)	3	75.0±1.4 [b] (n=2)
I. ric	LL	2	31.0±2.8 (n=5)	2	44.5±2.1 (n=2)	2	48.0±13.6 [j,i] (n=5)	2	44.5±2.1 (n=2)	-	-	-	-
	NN	2.7±0.5	44.5±1.0 [c] (n=9)	3	37.3±4.5 [c] (n=6)	3	34.0±6.3 [g] (n=5)	2.8±0.4	38.2±6.6 [d,l] (n=6)	3	95.0±5.4 [d] (n=10)	3	72.0±2.8 [d] (n=4)
Hybrid (♀*I.ric* × ♂*I.pers*)	LL	2	31.2±0.4 [e] (n=5)	2	39.7±4.6 [e] (n=3)	2	47.5±10.3 [e,h,k] (n=6)	3	51.0±1.4 [e] (n=2)	-	-	-	-
	NN	3	27.5±2.1 (n=2)	3	47.0±2.8 (n=2)	3	57.0±1.4 (n=2)	-	-	-	-	-	-
Hybrid (♀*I.pers* × ♂*I.ric*)	LL	2.7±0.6	37.0±1.4 (n=2)	2.3±0.6	42.7±9.8 (n=3)	2.3±0.6	100.7±17.9 [i,k] (n=3)	-	-	-	-	-	-
	NN	3	44.5±3.5 [f] (n=2)	3	53.0±5.7 [f] (n=2)	3	72.7±19.6 [f,g] (n=6)	3	61.5±9.1 [l] (n=8)	-	-	-	-

"-" no data; *I. ric – Ixodes ricinus*; *I. pers – Ixodes persulcatus*; mean ± SD is presented where applicable; [#] the beginning of molting was recorded for a group of ticks – about 100 larvae or 10-15 nymphs; superscript letters indicate statistically significant differences between groups according to Mann-Whitney U-test (2 groups) or Kruskal-Wallis H-test (3 and more groups) ($p<0.05$)

Table S3. The beginning of molting* of proto-female and proto-male nymphs of *Ixodes ricinus, Ixodes persulcatus,* and their hybrids under laboratory conditions

Species	Feeding in March, April, May		Feeding in June, July		Feeding in September		Feeding in October		Feeding in November	
	Females	Males	Females	Males	Females	Males	Females	Males	Females	Males
I. persulcatus	41.5±2.4 (n=4)	38.8±4.9 (n=4)	33.5±2.1 (n=2)	32 (n=2)	38±4.2 (n=2)	39 (n=2)	87 (n=1)	87 (n=1)	95 (n=1)	94 (n=1)
I. ricinus	44.7±0.6 (n=3)	44.3±1.5 (n=3)	38±4.2 (n=2)	32 (n=1)	39 (n=1)	36 (n=1)	39.3±6.0 (n=3)	37.0±8.2 (n=3)	97.0±7.0 (n=3)	95.0±7.0 (n=3)
Hybrid (♀*I.pers* × ♂*I.ric*)	-	-	-	-	82.7±25.6 (n=3)	62.7±2.1 (n=3)	65.3±8.1 (n=4)	57.8±9.5 (n=4)	-	-

"-" no data; mean ± SD is presented where applicable; * the beginning of molting was recorded for a group of ticks – about 100 larvae or 10-15 nymphs
